# Effects of a Home-Based Exercise Program on Health-Related Quality of Life and Physical Fitness in Dementia Caregivers: A Randomized Controlled Trial

**DOI:** 10.3390/ijerph19159319

**Published:** 2022-07-29

**Authors:** Josué Prieto-Prieto, Miguel Madruga, José Carmelo Adsuar, José Luis González-Guerrero, Narcís Gusi

**Affiliations:** 1 Department of Didactic of Musical, Plastic and Body Expression, School of Education and Tourism, University of Salamanca, 05003 Ávila, Spain; 2 Department of Didactic of Musical, Plastic and Body Expression, Faculty of Sport Sciences, University of Extremadura, 10071 Cáceres, Spain; miguelmadruga@unex.es (M.M.); jadssal@unex.es (J.C.A.); ngusi@unex.es (N.G.); 3 Geriatric Service, Complejo Hospitalario de Cáceres, 10004 Cáceres, Spain; joselglezg@gmail.com

**Keywords:** home-based exercise program, family caregivers, fitness, quality of life, dementia

## Abstract

Regular exercise can be an effective health-promotion strategy to improve the physical and mental health of informal caregivers. A randomized controlled trial study was designed to evaluate the effects of a 9-month home-based exercise intervention on health-related quality of life (HRQoL) and physical fitness in female family caregivers of persons with dementia. Fifty-four female caregivers were randomly assigned to two groups for the 9-month study period. Participants of the intervention group (*n* = 25) performed two 60-min exercise sessions per week at home, under the direct supervision of a personal trainer. Participants in the control group (*n* = 23) continued their habitual leisure-time activities. HRQoL was assessed using the SF-36 questionnaire, and physical fitness was measured using a battery of appropriate fitness tests. After 9 months, significant improvements were observed in general health, social function, vitality, hand and leg strength, trunk flexor and extensor endurance, and aerobic endurance in the intervention group. The present intervention was highly adherent and safe for the participants, with no dropout related to the intervention. As a home-based exercise program conducted by a personal trainer face to face, it can be considered as a feasible and appropriate method to improve the most deficient HRQoL dimensions and contribute to preserving the functional capacity of female family caregivers of persons with dementia.

## 1. Introduction

Dementia has been recognized as a major public health issue for healthcare systems, given the increasing number of people with this disease worldwide [1] and the economic implications of managing this disabling condition [2]. A majority of patients with dementia are cared for at home by a relative, who has the main role of providing support and help to care recipients [3,4,5,6]. These carers, who provide unpaid or informal attentions, play an essential role for the persons they are taking care of as well as for the overall healthcare system [7].

Research indicates that providing care to a person with dementia has negative physical, psychological, and social consequences for the caregiver [8,9,10,11,12]. In fact, these effects tend to be more severe in caregivers of people with dementia than those experienced by caregivers of non-dementia persons [13,14,15]. Previous studies have essentially focused on investigating the impacts of caregiving on mental health, such as increased burden, stress, mood disorders, anxiety, depression, emotional strain, and sleep problems [12,16,17,18,19,20,21,22]. However, this hazardous task has also been associated with a negative impact on physical health, leading caregivers to suffer from physical illness and disability [23] and from a higher risk of early mortality [24].

In addition, previous studies have identified an association between developing usual care tasks and an increased risk of physical diseases, such as arterial hypertension [25,26], an elevated blood pressure response [27], and reduced lymphocyte sensitivity and lowered cellular immunity [28], as well as coronary heart disease [29,30,31,32]. In addition, caregivers suffer from back problems, fractures, arthritis, and fatigue frequently [16,33,34,35] and have presented elevated obesity rates and a high level of sedentarism, which may substantially contribute to the high rate of obesity-related chronic conditions [36]. All these impairments significantly affect the caregivers’ capability to perform the usual care activities properly, and they are at higher risk of suffering health problems than non-caregivers [32]. All these facts underscore the need to carry out interventions on caregivers of persons with dementia to improve health-related physical fitness components in addition to mental health.

In this sense, regular physical exercise can be an effective health-promotion strategy to improve the physical and mental health of informal caregivers [37,38]. Physical-exercise-based interventions have reported positive outcomes for caregivers on quality of life, stress, burden, depressive symptoms, positive affect, self-efficacy, psychological responses to emotional challenges, sleep quality, metabolic risk, and physical fitness [39,40,41,42,43,44,45,46,47,48,49,50,51,52]; however, there is a lack of research that analyzes the effects physical exercise interventions have on specific fitness parameters.

Given that caregiver interventions are more likely to be effective if they are more accessible for participants [53], particular barriers of carers must be considered in exercise-based interventions, such as inability to leave the care recipient on their own or lack of time for leisure activities [54,55]. Besides the above-mentioned difficulties, other limitations have also been evidenced, such as the lack of flexibility and the long distances to regular services of physical activities in the community, which limit the opportunities of caregivers to be engaged in center- or community-based exercise interventions [56,57,58]. Consequently, home-based physical exercise interventions have been considered a tailored strategy for participating in health-promoting activities for family caregivers of persons with dementia [59].

Nonetheless, there is limited research evaluating this type of intervention, and a majority of them, after being prescribed, are supervised by telephone [40,42,44,57]. Adherence to home-based exercise programs supervised by telephone also requires a high degree of motivation from the individual [60], which would indicate that this may not be suitable in the dementia caregiver population, since they commonly experience apathy or a lack of motivation [61]. Therefore, an intervention performed at home under the instruction of a personal trainer could not only improve treatment adherence but can also involve both caregivers and care recipients [62].

Thus, the objectives of the study were the following: (a) to examine whether a 9-month individualized, home-based exercise program may have effects on quality of life and increase physical fitness levels in those caregivers; (b) to evaluate adherence rates to exercise-based programs in family caregivers of persons with dementia.

## 2. Materials and Methods

### 2.1. Trail Design and Participants

This study followed a between-subject experimental design on the basis of a randomized controlled trial (RCT). The participants were randomly assigned to one of the two groups: intervention group (IG) or control group (CG). This paper was developed in the framework of a large study to assess the cost utility of the intervention to the policymakers of the healthcare system. The required sample size was estimated into 50 caregivers distributed into two groups, IG and CG, as described in the study protocol [63]. Thus, the sample size calculation was estimated using the Spanish version of the EQ-5D-3L questionnaire and considering the between-subject experimental design of the study. A significance level alpha of 0.05 and statistical power of 80% were assumed, which are required to detect a minimal clinically significant difference of 0.20 and SD of 0.12 [64] to know if the intervention could be a cost-effective addition to usual care of the caregivers studied. More comprehensive information about the sample size is explained in the study protocol [63].

Participants were recruited in cooperation with the regional associations of relatives of patients with dementia from Extremadura (Spain). Potential participants were screened for eligibility in accordance with the study inclusion and exclusion criteria. The inclusion criteria were: being a female primary informal caregiver for a relative with dementia, for whom diagnosis was performed by a qualified physician; living at home with the patient with dementia; providing at least 20 h of in-person care per week [33]; being age 50 years or older and not having any medical condition that would limit participation in moderate-intensity exercise, as assessed by the Physical Activity Readiness Questionnaire [65]; no participation in any regular physical activity program (i.e., less than two ≥ 20-min sessions of exercise per week during the previous 6 months before the intervention); having no changes in medication for at least 3 months prior to study entry; and not having plans to move from the place of residence within the following 12 months. The exclusion criteria were the opposite criteria plus having any external economic or personal support for caregiving of the patient with dementia.

**Figure 1:**
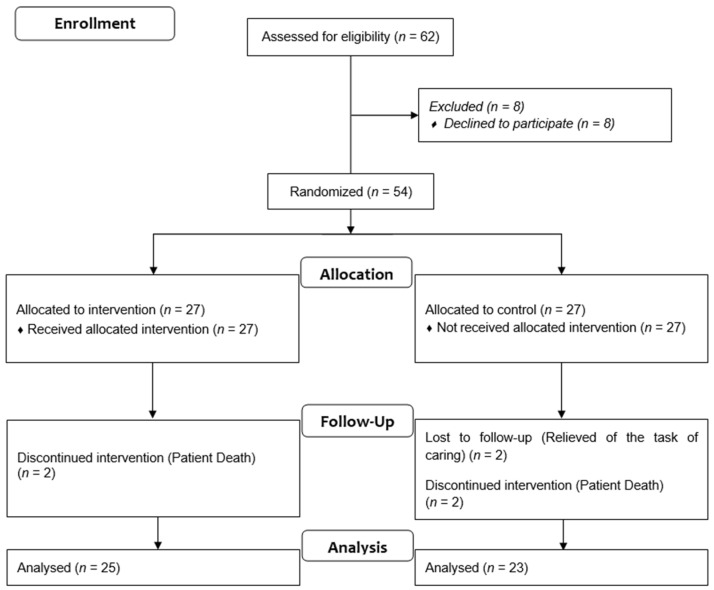
Outline of research process.

After sending invitations to participate in the study to all potential participants, 62 subjects accepted to participate in the study. After the screening, 8 female informal caregivers were excluded from the study. Moreover, data of six subjects were excluded from the statistical analyses for the following reasons: (1) death of the care recipient (*n* = 4); (2) care recipients’ change of residence and subsequent changeover of the caregiver (*n* = 2). Therefore, a final total of 48 participants were randomly assigned to either the IG (*n* = 25) or to the CG (*n* = 23) (see Figure 1). All participants signed a written informed consent form before participating in the study. The study was conducted in accordance with the updates of the Declaration of Helsinki (ISCRCTNS80414567).

### 2.2. Intervention

Participants of the IG completed a 9-month-long and home-based physical exercise intervention twice a week for 1-h session at their homes. This intervention was supervised by a qualified personal trainer, who conducted face-to-face exercise sessions. Prior to the start of the exercise intervention, and to standardize the session performance, the personal trainers were instructed within 2-session workshop on how to appropriately apply the exercises during the session. Physical exercise intervention basically consists of aerobic exercises of moderate intensity, which means about 3 to 6 metabolic equivalents. Each session comprised 10-min-long warm-up activities, in particular slight movements of progressive intensity and easy walks and steps; 10 min of lasting aerobic exercise at 60–65% of maximal heart rate (HR_max_); 20 min of overall mobility and strength exercises using the weight of one’s own body; additional 10 min of lasting aerobic exercise at 60–65% HR_max_; and 10 min of lasting cool-down with low intensity exercises.

During each session, participants wore a heart rate monitor to continuously monitor their heart rate to ensure that the caregivers were performing the appropriate intensity (Polar S625X, Polar Electro Oy, Kempele, Finland). The heart rate was set by protocol, and data from heart rates were not collected or used as either dependent or independent variables of the outcome. In order to enhance participants’ enjoyment of the exercises and fitness levels, materials such as weights, dumbbells, and elastics were included into the basic exercises. The progression of training was based on the increase of absolute effort for the same percentage of loads and the complexity of movements during aerobic activities according to individual capacities.

Throughout the 9-month-long study, participants of the CG were instructed to continue their normal daily activities and not to start any new physical exercise programs, to avoid influencing the study. In addition, during the 9-month-long intervention period, participants of the CG were contacted once a month by telephone to conduct an ad hoc standardized, non-exercise-related conversation with a member of the research team, in order to remind them of the conditions of the study.

### 2.3. Measures

Participants of both groups were evaluated at home by two qualified and trained members of the research team (blind to treatment allocation), at baseline and at the end of the intervention. In order to apply a standardized evaluation protocol, prior to the baseline evaluation, the evaluators received a testing manual that was ad hoc written by the research team. This manual contained the descriptions and particular instructions for all assessment procedures. They also participated in an assessment training workshop that consisted of three sessions, each 3–4 h long. All evaluation measures have been shown to have moderate to high reliability and validity in samples of older people and caregivers. The duration of each evaluation session was approximately 45 min, and it was organized in two phases. Participants first completed an ad hoc-made questionnaire with sociodemographic and caregiving-related characteristics, subjective burden, and health-related quality of life outcomes. In the second phase, participants performed a battery of health-related fitness tests.

*Socio-demographic and caregiving-related characteristics.* Data compiled included age, place of residence, marital status, number of co-resident persons, educational level, smoking and alcohol consumption habits, and level of physical activity. It also included questions regarding the nature of the relationship between the primary caregiver and the care recipient, as well as the number of years that the caregiver spent caring for the care recipient. Caregiver´s subjective burden was assessed using the Zarit Burden Interview [66]. This 22-item self-report questionnaire examines the burden experienced by caregivers on the basis of functional, psychological, behavior, and economic factors. The scoring is conducted on a scale of 0–88, with higher scores reflecting higher burden. The Spanish version of the Zarit Burden Interview was validated and adapted by Martin et al., in 1996. It establishes the following cut-off scores: 22–46, no burden; 47–56 moderate burden; and 57–110, severe burden [67]. Regarding the care recipients, functional status was evaluated by trained members of the research team using the Barthel Index [68]. This 10-item tool assesses the care recipient’s capacity to perform 10 basic activities of daily life, such as the ability to dress or bathe. Numerical scores are assigned according to whether the individual requires assistance to perform the task or whether he/she is able to perform the activity independently. The final score provides a quantitative estimation of the care recipient’s level of dependency, ranging from 0 to 100. Higher scores indicate a higher level of functional independence, and the opposite pattern is indicated by lower scores.

*Health-related quality of life (HRQoL).* HRQoL was measured using the validated Spanish version of the *Short Form 36 health survey questionnaire (SF-36)* [69,70]. This is a generic measure that includes 36 items and involves 2 dimensions of physical component summary (PCS) and mental component summary (MCS) and 1 health transition item (HT). The PCS contains 4 parameters: physical function (PF), physical role limitations (RP), bodily pain (BP), and general health (GH). The MCS contains 4 parameters: vitality (VT), social functioning (PS), emotional role limitations (RE), and mental health (MH). The scores for each dimension and parameter range from 0 (worse health state) to 100 (best health state).

*Physical fitness assessments*. Participants completed a battery of health-related fitness tests for older people over a period of approximately 45 min. The participants were tested without an initial warm-up period to control or reduce any resultant individual variability. Weight, height, and the circumference of the waist and hips were measured in accordance with the recommendations of the European Council [71] for calculating the *body mass index* (BMI) and the *waist–hip ratio* (WHR). *Hand-grip strength* was assessed in both hands using a hand dynamometer (TKK, Tokyo, Japan) [72]. Outcome was defined as the mean value for both hands. *Lumbar trunk muscle endurance* was assessed using two tests [73]. To evaluate flexor endurance, the participant was asked to lie in a supine position and to raise the lower extremities with 90° flexion of the hip and knee joints. To evaluate extensor endurance, the participant was asked to lie in a prone position while holding their sternum off the floor. During both procedures, the participant was asked to maintain the original position for as long as possible within a 2-min time limit. Outcome was defined as the time (in seconds) over which the participant could maintain each position. *Flexibility* was assessed using the sit-and-reach test [74]. During this trunk flexion, the distance between the tips of the fingers at the starting and final positions was recorded. Outcome was defined as the best result of three trials. *Lower-extremity function* was assessed using the chair-stand test [75]. The participant was seated in a standardized chair (43.0 cm in height) and asked to fold her arms across her chest. She was then asked to stand up and then sit back down again 10 times as quickly and as safely as possible. Outcome was defined as the best result of two trials (expressed in seconds). *Mobility* (speed, agility, and balance) in that context was assessed by using the timed up-and-go test [76]. This test involves rising out of a chair, walking 3 m to and around a cone, and returning to the chair in the shortest time possible. The best score of two trials was recorded*. Balance* was measured by administering the functional reach test [77]. In this test, the maximum distance that one can reach forward beyond arm’s length while standing and maintaining a fixed base of support (feet placed shoulder width apart) is tested. The best score of two trials was recorded. *Maximal oxygen uptake (VO2 max)* as a parametric measure of cardiorespiratory fitness was assessed using the Canadian Aerobic Fitness Test [78]. This test involves a progressive, submaximal aerobic protocol, in which the participant is asked to step up and down a double step (40.6 cm) at a rhythm determined according to the participant’s age and sex. Stepping was performed with a six-pace cycle: one foot on the middle step, both on the top step, one on the middle step, and both feet on the ground. The test was preceded by a specific 3-min warm-up. This was followed by three stages. Each stage lasted for 3 min and was performed at a different stepping rate. Heart rate was continuously monitored every five seconds using a pulse meter (Polar S625X, Polar Electro Oy, Kempele, Finland) to ensure that it remained within a specific “safety” zone. If the heart rate at the end of the first stage was within safe limits, the participant was allowed to progress to the second stage. After a further 3 min of stepping, the heart rate was re-measured. If the “safety” limit had still not been reached, the participant was allowed to progress to the third stage, which involved a higher rate of stepping. Outcome was defined as VO2 max, which was estimated on the basis of test performance level, heart rate, age, and gender.

*Exercise adherence.* Each participant in the intervention group was offered a total of 72 exercise sessions. The supervising personal trainer recorded the number of sessions completed during the 9-month-long period. Exercise adherence rates were calculated on the basis of the percentage rate of completed sessions.

### 2.4. Statistical Analysis

Descriptive analyses were conducted to evaluate between-group differences at baseline. For this analysis, Student’s *t*-test for continuous variables and Pearson’s chi-squared test for categorical variables were used. In addition, the Kolmogorov–Smirnov with the correction of Lilliefors test was also used to test normal distribution of variables. Moreover, an analysis of variance was performed to evaluate between-subject differences at baseline and at the end of the 9-month-long intervention. This analysis was, additionally, adjusted for several factors that could act as potential mediators of the changes in the measured variables, such as caregiver’s age and functional dependency of the care recipient (using the Barthel Index) at baseline. In addition, the effect sizes (*d* of Cohen) for the standardized mean differences between the IG and the CG were also included: 0.20–0.49 for small effect; 0.50–0.79 for medium effect; and equal to or larger than 0.80 for large effect [79]. Finally, analysis of variance for repeated measures with Bonferroni adjustment was performed to evaluate potential differences between the IG and the CG after the intervention. According to Hunter and Schmidt [80], this analysis helps increase precision of resulting data.

For all tests, the significance level was defined as *p* < 0.05.

## 3. Results

### 3.1. Sociodemographic and Caregiving-Related Characteristics of Participants

Baseline analysis indicates no significant differences between the IG and the CG on either sociodemographic or caregiving-related characteristics (Table 1). The mean age of the 48 participants who were enrolled in the study was 60.17 years (SD = 8.49). A total of 81.25% (39/48) of the participants were caring for a relative with Alzheimer’s disease, 14.58% (7/48) were caring for a relative with stroke-related dementia, and 4.16% (2/48) were caring for a relative with other types of dementia. Moreover, all participants reported having been involved in the caring role for more than 5 years. A total of 75% were living at home with the care recipients, and 68.8% were living in an urban area. The participants reported having spent 42 to 168 h per week engaged in caregiving duties.

### 3.2. Intervention Adherence

A total of 22 out of 25 participants in the IG (88%) completed up to 90% of the physical exercise sessions offered. There were no reported adverse events (health-related problems or secondary discomforts) during the home-based intervention. The rest of the participants in the IG (12%) completed up to 80% of the sessions offered. In addition, these participants of the IG completed 62 to 72 sessions (69.7 ± 3.4), which means an intervention adherence of up to 90%. The main reason for the few missed sessions by caregivers was the need to attend, together with the care recipient, an appointment with their healthcare provider.

### 3.3. Effects of Intervention

Primary outcome scores at baseline and at the end of the 9-month intervention for the IG and CG are shown in Table 2 and Table 3. There were no between-subject differences on the dimensions of HRQoL nor in fitness parameters at baseline. At the end of the 9-month intervention, significant improvements within the IG in HRQoL were found, specifically in the dimensions of general health (mean difference 6.96, CI 95% 0.13 to 13.79), vitality (mean difference 8.40, CI 95% 2.69 to 14.11), and mental health (mean difference 8.64, CI 95% 2.91 to 14.37), whereas in the CG, in measures of HRQoL, no significant differences were found. Unadjusted mean difference between-group effects were significant (*p* < 0.05) in general health, vitality, and social functioning but not in mental health. In addition, medium effects (*d* of Cohen) were found in those variables. No significant differences were observed within and between groups in the physical and mental component summary of the SF-36. These results are also shown in Table 2.

Regarding health-related fitness, participants in the IG showed a significant improvement compared with the baseline in flexor trunk endurance (34.04 95% CI 17.13 to 50.95), extensor trunk endurance (38.03 95% CI 22.59 to 53.48), leg strength (−7.04 95% CI −9.49 to −4.58), functional reach (3.38 95% CI 0.36 to 6.40), and aerobic endurance (2.56 95% CI 1.34 to 3.79). The CG showed a significant decline in flexibility (−2.30 95% CI −4.12 to 0.49). After the intervention, mean changes in waist–hip ratio were significant in both groups, whereas in the IG it increased by 0.04 (95% CI 0.00 to 0.79), and in the CG it decreased by −0.08 (95% IC −0.01 to −0.00). Unadjusted mean difference between-group effects were significant in weight, waist–hip ratio, bimanual strength, flexor trunk endurance, extensor trunk endurance, leg strength, and aerobic endurance. In addition, medium and large effects (*d* of Cohen) have also been shown in those fitness measures. Particularly, the waist–hip ratio in participants of the IG increased to >0.85, which could represent a cardiovascular risk, and the BMI score increased up to 28.3, which could be considered a cardiovascular risk as well [81,82]. After adjusting by age and Barthel Index, there were significant differences in all variables mentioned above and also in functional reach. These results are shown in Table 3.

## 4. Discussion

The main findings of this randomized controlled trial were that a 9-month home-based exercise program was feasible and effective at improving HRQoL and physical fitness in female family caregivers of people with dementia. There are limited trials in this population that have evaluated the effects of structured physical exercise interventions, especially those conducted by a personal trainer face to face at home, so our findings add some important new information to the current literature.

The results are consistent with previous research, which has indicated that an exercise program for caregivers of people with dementia can improve physical health and mental symptoms [37,38]. Previous studies have essentially focused on mental outcomes, such as burden, anxiety, depression, and other psychological symptoms, and the interventions that assessed physical parameters were minimal. In the current study, the most relevant improvements in HRQoL, assessed by the SF-36, were found in mental health dimensions, specifically in vitality and social functioning. Moreover, despite the lack of statistically significant between-group differences, a small/medium effect size on the mental health dimension was demonstrated in the intervention group. This is a noteworthy finding, given that mental health dimensions have been reported to be adversely affected in HRQoL in dementia caregivers compared with population norms [35,83,84,85].

With regard to physical health dimensions, significant effects between the IG and the CG after 9 months were observed in the dimension of general health of the SF-36. This finding could be, at least partially, explained due to the characteristics of participants in the study, as they had high baseline physical component scores, especially on physical functioning, meaning that further improvements would be difficult to detect. This fact is consistent with previous studies, where caregivers only reported better scores than non-caregivers for the dimensions related to physical functioning [35]. This is probably due, at least in part, to the daily physical activity involved in managing a patient with dementia, such as seating the patient or lifting them up.

As hypothesized, at the end of the 9-month-long intervention, participants in the intervention group showed an improvement in fitness measures. These effects were statistically significant in strength and endurance, which are essential aspects for caregivers to provide effective care. In particular, muscular endurance of the muscles of the trunk improved significantly in caregivers of the IG vs. CG. This finding is potentially important because caregivers usually suffer from back pain caused by caring tasks [35], and this fitness component could be tightly associated with that impairment. In addition, despite the program design not being based on specific endurance physical activities such walking, treadmill, cycling, etc., the program was effective at improving cardiorespiratory fitness, measured by VO2max, which is a remarkable finding given the risk of coronary disease presented by caregivers [29,31].

Previous research on exercise-based interventions has essentially focused on evaluating the effects on mental outcomes, and few studies have measured changes in physical fitness [37,38]. Hill et al. [43] found improvements in balance, leg strength, and gait endurance after a 6-month center-based intervention (non-randomized) in caregivers of patients with undefined illnesses. They used several types of physical activity (Tai-chi, strength training, yoga, and circuit training), but results were not reported for each activity group. In accordance with findings from Hirano et al. [44] and King et al. [39], no significant reduction was observed for the BMI between groups. Nonetheless, in the present study, it was found that BMI increased slightly in the IG and decreased in the CG. This finding could be explained by the following factors. Significantly improved strength scores were observed in the IG, thus, the increase in the BMI might be associated with the gain in lean body mass and the weight increase, however, muscle mass could not be assessed in this study [86]; however, those associations were not evaluated. Moreover, regular physical exercise can counteract body composition changes with aging, especially over 60–70 years old [87], and may be due to a loss of muscle mass, bone, or organ tissue rather than to a loss of body fat [88,89].

A major strength of the current study was the feasibility of this type of program. The intervention was well accepted, with 89% of caregivers completing the 9-month program with no voluntary dropouts. This is an important finding considering the reported barriers for caregivers to be engaged in regular physical activity or exercise programs [56,57,58,90]. Moreover, the intervention was found to be safe, with no participants reporting an adverse event.

Adherence rate was remarkably high, as caregivers of the IG attended 97% of sessions. Attendance in our intervention is at the highest adherence rates reported in previous studies, both in center-based and home-based exercise programs [37,38], even in comparison with shorter interventions developed at home [50]. To our knowledge, the present study is unique in that it used such an extended intervention with direct supervision face to face at caregivers’ homes, as other longer interventions were supervised by telephone or video [39,42,43,55].

Several factors could account for the high adherence observed in the present study. One facilitating factor is the relative flexibility of the program, which has been personalized according to caregivers’ demands and context by considering the lack of free time or flexibility experienced by these caregivers in attending programs outside the home due to family and caring responsibilities [44,91,92,93]. Finally, another motivating factor could be the direct interaction with the personal trainer, which allows for coping with the apathy or lack of motivation that family caregivers commonly experience when trying to engage with or adhere to an exercise program, as well as their limitations to devote leisure time to exercise [90].

To sum up the relevance of this study, since there is limited research evaluating the effects of individualized physical exercise interventions on caregivers’ fitness, this study elucidates that practicing supervised physical exercise regularly and at home may have positive effects for fitness and HRQoL in family caregivers. In addition, this study highlights the importance of developing individualized and tailored interventions for this population, since, due to their caring responsibilities, they have several limitations to participate in center- or community-based exercise interventions [56,57,58]. Thus, this intervention was effective, as it was adapted to the characteristics of family caregivers.

It is also important to highlight that the present study has several limitations. First of all, although the family caregivers exhibited the characteristics described in previous studies of this population in Spain [33], the conclusions must be applied with caution when considering individuals with different characteristics, e.g., those who are male or from other age groups or who have a different previous level of physical activity, number of hours of providing care, or caregivers receiving assistance or other support from another caregiver, with a low to medium income, while caregivers with greater financial resources tend to employ a professional formal caregiver to assist in the provision of care.

A further limitation was the sample size. This may have limited the statistical power to detect changes in certain variables.

Moreover, this research does not elucidate how the effects of exercise can be maintained over a longer period after completion of the home-based intervention; thus, a follow-up period should be added in future studies.

In addition, despite participants being instructed not to perform any physical activity for >20 min two days a week and due to the design of the study and the available resources, we were not able to monitor leisure-activity movements during the 9-month study. Thus, this could also be considered a limitation of this study.

Finally, the cost of implementing this program can be expensive and may vary between rural and urban areas. Further studies to compare the efficacy and cost-effectiveness of several approaches are warranted, such as exercise program interventions delivered via the Internet, videoconferencing, or by volunteers.

## 5. Conclusions

A home-based exercise intervention that includes twice-weekly sessions directed by a personal trainer improves HRQoL and contributes to preserving the functional capacity of female family caregivers of persons with dementia, particularly in such fitness measures as strength, endurance, balance, and VO2max. Despite these promising results, the optimal dose–response requires new specific research designs to compare different exercise interventions (e.g., number of sessions per week and exercise load per session). It is also recommended that specifically tailored exercise interventions be developed, including hand strength, flexibility, strength of trunk and legs, and endurance, among the support services offered to caregivers of persons with dementia, to maintain or promote physical and mental health.

The present intervention has also been shown to be highly adherent and safe for the participants; therefore, it can be considered as a feasible and appropriate method to help female family caregivers to improve their health status.

Finally, future studies should be performed to analyze other home-based interventions, such as web-based or interactive physical exercise programs, to determine the most suitable intervention for family caregivers. Individualized interventions may be the most effective for family caregivers at home; however, more resources and personnel are required. Thus, future research into the cost-effectiveness of this home-based exercise intervention should be conducted.

## Figures and Tables

**Table 1:** Sociodemographic and caregiving-related characteristics of participants at baseline.

	All (*n* = 48)	Intervention (*n* = 25)	Control (*n* = 23)	*t*-Test/χ^2^	*p* ^1^
	Mean (SD)	Mean (SD)	Mean (SD)		
Age (years)	60.17 (8.49)	60.96 (8.99)	59.30 (8.01)	−0.671	0.505
Age of patient (years)	79.85 (7.92)	79.44 (10.12)	80.30 (4.67)	−0.385	0.703
Caregiver years	5.48 (3.31)	5.28 (2.94)	5.70 (3.72)	−0.431	0.668
Diagnostic years of the patient	6.94 (4.17)	7.00 (4.58)	6.87 (3.77)	−0.107	0.915
Zarit Subjective Burden	55.73 (14.31)	55.72 (14.21)	55.74 (14.74)	−0.005	0.996
Barthel Index patient	31.81 (29.64)	31.68 (26.38)	31.96 (33.43)	−0.032	0.975
	(*n*, %)	(*n*, %)	(*n*, %)		
Alzheimer’s disease	39 (81.3)	21 (84.0)	18 (78.3)	0.291	0.865
Relative: daughter	28 (58.3)	13 (52.0)	15 (65.2)	0.894	0.639
Living with patient	37 (77.1)	20 (80.0)	17 (73.9)	0.251	0.616
Living in urban areas	33 (68.75)	20 (80.0)	13 (56.5)	3.074	0.080
Marital status: married	35 (72.9)	17 (68.0)	18 (78.3)	2.950	0.566
Living with husband	36 (75.0)	17 (68.0)	19 (82.6)	2.699	0.259
Education: primary school	29 (60.4)	16 (64.0)	13 (56.5)	3.600	0.308
Not smoker	42 (87.5)	24 (96.0)	18 (78.3)	4.782	0.188
Not alcohol consumer	35 (72.9)	19 (76.0)	16 (69.6)	5.183	0.394
Physical inactivity ^2^	35 (72.9)	16 (64.0)	19 (82.6)	10.525	0.161

^1^ *p*-values of analysis of *t* or χ^2^. ^2^ Physical inactivity includes respondents who never walk at least 30 min a day.

**Table 2:** HRQoL at baseline and after 9-month intervention.

Outcomes	Group	Baseline Mean (SD)	9 Months Mean (SD)	Between-Group Effect Mean (95% IC)	Effect Size ^1^	*p* ^2^	*p* ^3^	*p* ^4^
Physical functioning	Intervention	81.20 (14.59)	84.60 (17.13)	3.62 (−4.54 to 11.78)	0.19	0.377	0.405	0.381
	Control	80.43 (22.10)	80.22 (21.50)					
Physical role limitations	Intervention	59.00 (40.10)	62.00 (43.97)	0.83 (−18.88 to 20.53)	0.02	0.933	0.718	0.936
	Control	68.47 (39.32)	70.65 (38.18)					
Bodily pain	Intervention	66.48 (22.96)	64.36 (25.89)	0.31 (−14.41 to 15.04)	0.01	0.966	0.857	0.966
	Control	60.47 (32.29)	58.04 (30.26)					
General health	Intervention	57.16 (17.49)	64.12 (16.90)	8.70 (0.39 to 17.00)	0.43 *	0.040	0.029	0.043
	Control	57.39 (21.91)	55.65 (19.19)					
Vitality	Intervention	54.80 (18.96)	63.20 (18.53)	8.18 (0.29 to 16.07)	0.42 *	0.042	0.054	0.035
	Control	50.22 (19.27)	50.43 (23.25)					
Social functioning	Intervention	73.91 (33.27)	78.26 (28.51)	−12.85 (−25.01 to −0.69)	0.43 *	0.039	0.045	0.040
	Control	83.00 (24.17)	74.50 (27.36)					
Emotional role limitations	Intervention	74.67 (38.82)	76.00 (36.67)	1.33 (−16.04 to 18.71)	0.03	0.878	0.900	0.875
	Control	66.67 (46.05)	66.67 (43.81)					
Mental health	Intervention	59.52 (16.54)	68.16 (15.81)	7.60 (−0.09 to 15.28)	0.39	0.053	0.071	0.052
	Control	56.17 (21.79)	57.21 (20.45)					
SF-36 PCS	Intervention	46.44 (7.67)	46.85 (9.67)	0.80 (−4.08 to 5.98)	0.01	0.743	0.846	0.724
	Control	47.40 (8.60)	47.02 (9.64)					
SF-36 MCS	Intervention	44.21 (11.82)	46.04 (12.19)	0.91 (−4.43 to 6.25)	0.07	0.733	0.578	0.747
	Control	40.29 (13.95)	41.21 (12.86)					

^1^ *d* of Cohen. ^2^ *p*-value of analysis of variance to compare differences between groups at 9 months. ^3^ *p*-value age adjustment of analysis of variance to compare differences between groups at 9 months. ^4^ *p*-value Barthel adjustment of analysis of variance to compare differences between groups at 9 months. * *p* < 0.05.

**Table 3:** Physical fitness at baseline and after 9-month intervention.

Outcomes	Group	Baseline Mean (SD)	9 Months Mean (SD)	Between-Group Effect Mean (95% IC)	Effect Size ^1^	*p* ^2^	*p* ^3^	*p* ^4^
Weight (Kg)	Intervention	69.19 (18.26)	70.41 (18.31)	2.03 (0.12 to 3.95)	0.13 *	0.038	0.042	0.039
	Control	67.50 (12.07)	66.68 (12.04)					
BMI (kg·m^−2^)	Intervention	27.9 (6.73)	28.2 (6.69)	0.69 (−0.08 to 1.45)	0.12	0.077	0.087	0.080
	Control	27.6 (5.12)	27.2 (4.86)					
Waist–Hip ratio	Intervention	0.814 (0.064)	0.856 (0.100)	0.05 (0.01 to 0.09)	0.76 *	0.012	0.014	0.011
	Control	0.843 (0.063)	0.836 (0.062)					
Bi-handgrip strength (kg·m^2^)	Intervention	45.46 (12.16)	47.18 (9.95)	3.22 (0.29 to 6.17)	0.31 *	0.033	0.038	0.030
	Control	44.89 (7.64)	43.39 (8.46)					
Endurance flexor trunk (s)	Intervention	31.46 (25.09)	65.49 (43.29)	31.81 (12.27 to 51.35)	0.92 **	0.002	0.002	0.002
	Control	35.33 (41.77)	37.56 (40.06)					
Endurance extensor trunk (s)	Intervention	44.19 (44.77)	82.23 (42.85)	39.86 (19.45 to 6.27)	0.84 ***	<0.001	<0.001	<0.001
	Control	45.70 (48.66)	43.87 (48.42)					
Flexibility (cm)	Intervention	24.84 (9.79)	25.04 (9.48)	2.50 (−0.51 to 5.52)	0.27	0.101	0.126	0.105
	Control	23.59 (8.29)	21.29 (9.05)					
Leg strength (s)	Intervention	22.69 (6.63)	15.66 (5.77)	−7.28 (−11.56 to −3.00)	−0.89 **	0.001	0.001	0.001
	Control	19.74 (9.26)	19.99 (9.48)					
Time up and go (s)	Intervention	6.29 (1.39)	6.05 (2.28)	−0.74 (−1.90 to 0.41)	−0.31	0.203	0.196	0.205
	Control	7.34 (3.07)	7.84 (4.34)					
Functional reach (cm)	Intervention	29.78 (4.91)	33.16 (7.17)	3.08 (−0.37 to 6.52)	0.51	0.079	0.033	0.082
	Control	28.35 (6.91)	28.65 (7.07)					
PAC (VO2max)	Intervention	22.45 (3.78)	25.01 (3.37)	2.14 (0.62 to 3.65)	0.63 **	0.007	0.018	0.006
	Control	22.57 (2.79)	23.00 (2.57)					

^1^ *d* of Cohen. ^2^ *p*-value of analysis of variance to compare differences between groups at 9 months. ^3^ *p*-value age adjustment of analysis of variance to compare differences between groups at 9 months. ^4^ *p*-value Barthel adjustment of analysis of variance to compare differences between groups at 9 months. * *p* < 0.05; ** *p* < 0.01; *** *p* < 0.001.

## Data Availability

The data presented in this study are available on request from the corresponding author. The data are not publicly available due to the privacy policy of Alzheimer’s Family Associations.
